# Ureterocalyceal Fistula: A Rare Complication of Laparoscopic Partial Nephrectomy

**DOI:** 10.1155/2020/8827444

**Published:** 2020-09-29

**Authors:** Fayez T. Hammad

**Affiliations:** College of Medicine and Health Sciences, United Arab Emirates University and Mediclinic Al Ain, Al Ain, UAE

## Abstract

**Background:**

Postoperative urinary leak is a well-documented complication following partial nephrectomy. It usually presents as persistent discharge from the retroperitoneal drain, nephrocutaneous fistula, urinary collection, systemic manifestations, or abdominal symptoms. Herein, we report for the first time on a case of urinary leak postlaparoscopic partial nephrectomy which did not heal and led to the formation of ureterocalyceal fistula. *Case Presentation*. A 41-year-old male presented with a coincidental renal mass at the inferiomedial aspect of the right kidney. He underwent laparoscopic partial nephrectomy. On the third postoperative day, he developed fever. CT scan showed minimal urine leak from the tumor site and a JJ stent was inserted. Due to severe bladder symptoms, the stent was removed and a perirenal drain was inserted and removed in few days. He did well initially but in two weeks, he started to develop urinary tract infections. Repeat CT scan showed ongoing urinary leak from the site of the previous surgery. Retrograde pyelography demonstrated a complete UPJ stenosis with an ureterocalyceal fistula. Trial for reanastomosis failed due to severe adhesions and small intrarenal pelvis. An ureterocalyceal anastomosis has to be performed to another calyx.

**Conclusion:**

We report for the first time on an ureterocalyceal fistula following laparoscopic partial nephrectomy. This complication might be prevented by a careful dissection of the area close to the ureter or by an insertion of a JJ stent for an adequate time if a ureteric injury is suspected.

## 1. Background

Nephron-sparing surgery has become the standard of care for clinical stage T1a and selected clinical stage T1b renal tumors [1–3]. This is due to the superior functional outcomes and equivalent oncologic results compared with radical nephrectomy. Despite the advancement in technology, it remains a challenging procedure associated with several postoperative complications [4, 5].

Postoperative urinary leak following partial nephrectomy is a well-documented complication with an incidence ranging from 18% in some series to less than 2% in the most recent ones [4–6]. It usually presents as persistent discharge from the retroperitoneal drain, nephrocutaneous fistula, urinary collection, systemic manifestations, or abdominal symptoms [6–8].

Herein, we report for the first time on a case of urinary leak postlaparoscopic partial nephrectomy which led to the formation of a ureterocalyceal fistula.

## 2. Case Presentation

**Figure 1:**
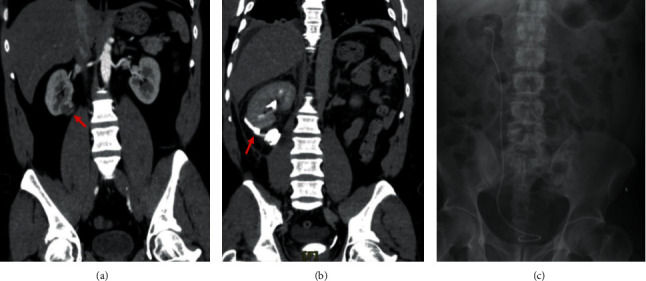
(a) Preoperative CT scan showing the lesion at the inferiomedial aspect of the right kidney. (b) Delayed phase CT scan demonstrating the urine leak from the site of partial nephrectomy. (c) Postoperative plain X-ray showing the JJ stent in position.

A 41-year-old male presented with a coincidental right renal mass. He gave a history suggestive of chronic prostatitis and had mild urinary frequency and occasional mild urgency. He also had a history of previous open appendectomy. Preoperative CT scan showed a heterogeneously enhancing mass at the inferior pole of the right kidney suggestive of neoplastic lesion (PADUA score 8) (Figure 1(a)) for which he underwent transperitoneal laparoscopic partial nephrectomy with 25 minutes warm ischemia time and the surgery was uneventful. During surgery, the right colon was reflected medially, followed by dissection of the renal hilum, and identification of the artery and vein. The tumor was identified and the resection margins were identified and marked with cautery. After clamping the renal artery, the tumor was resected with an approximately 3-5 mm rim of normal renal parenchyma. We could not identify any urinary leak and the resection bed was sutured using polyglactin in a running fashion. The renal capsule was approximated over a small piece of *Surgicel* using polyglactin as well. Histopathology demonstrated a 2.9 × 3.8 × 3.4 cm Fuhrman grade II clear cell carcinoma which was confined to the capsule (T1a N0 M0).

Postoperatively, he did well and the drain was removed after 48 hours as it was bringing only 45 cc of serosanguinous fluid as suggested by potassium and creatinine levels, which were similar to the serum values. One day later, he developed high-grade fever, and CT scan showed minimal urine leak from the site of the partial nephrectomy (Figure 1(b)). He refused nephrostomy tube insertion, so he underwent cystoscopy and JJ stent insertion on the 6th postop day. During the procedure, retrograde pyelography showed no abnormality in the upper ureter or kidney apart from the leak from the site of surgery. Therefore, 26 cm, 6 FR stent was inserted easily (Figure 1(c)) and a Foley's catheter was fixed. The patient was discharged on the eighth postop day.

Three days postdischarge, he presented with severe bladder spasms and insisted on catheter removal which was reinserted 24 hours later. He then developed fever with minimal abdominal pain for which he was admitted on the 9th postdischarge day. CT scan showed extravasation of urine at the site of the previous surgery with fluid collection (3.8 × 1.8 cm) associated with few gas bubble and mild right pleural reaction. He was offered a nephrostomy tube but he totally refused again, so he was convinced to have a perirenal drain, which was inserted in the presence of the JJ stent and urethral catheter. Subsequently, he continued to have severe bladder spasms despite the antimuscarinic agents. Indeed, he tried several times to pull the catheter which got blocked and all the urine from both kidneys was coming out from the perirenal drain. In view of this and the agony that he had, we decided to remove the JJ stent and urethral catheter and leave only the perirenal drain.

Subsequently, almost half of urine output was coming out from the perirenal drain. Another CT scan showed the pigtail drain tube to be adjacent to the site of partial nephrectomy and no contrast was going down the ureter. So, the drain was pulled out 2 cm under CT guidance. CT scan then showed free drainage of urine along the ureter. The drain was left in place for two days during which it did not drain any urine. The patient felt well with no fever, so the drain was removed three days later. During all this time, the kidney did not show any significant dilation on either ultrasound or CT scan.

**Figure 2:**
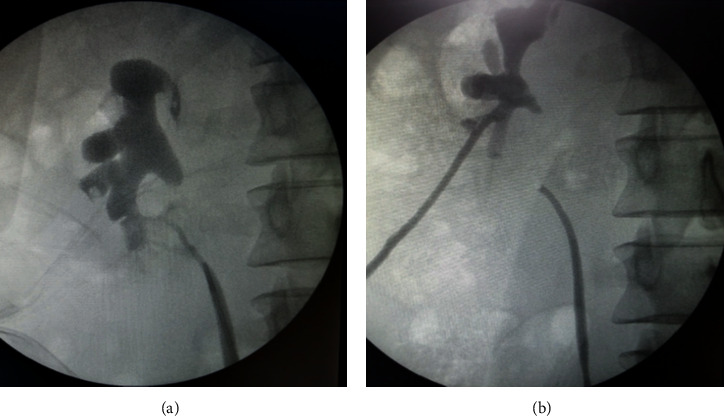
(a) Retrograde pyelography showing a fistulous tract between the upper ureter and the inferior calyceal system. The blind end of the renal pelvis is also seen. (b) Simultaneous retrograde and antegrade pyelography following the insertion of the nephrostomy tube.

Postdischarge, he did well initially, but in few days, he started to develop urinary tract infections that were occasionally associated with low-grade fever but no abdominal pain. Ultrasound again did not show any abnormality. One month later, he was readmitted with high-grade fever. CT scan showed an ongoing leak which was, this time, more medial with minimal dilation of the collecting system. A trial to insert a JJ stent failed and retrograde pyelography showed normal ureter up to approximately 3 cm below the ureteropelvic junction which had a blind end and there was a fistula between the upper ureter and lower calyceal system (ureterocalyceal fistula) (Figures 2(a) and 2(b)). Using an ureteroscope, a trial to negotiate the way to the UPJ was not successful. A nephrostomy tube was inserted and a trial to pass a JJ stent and guidewire, antegradely, also failed. Six weeks later, he had another trial to pass the stent antegradely which also failed. He then underwent an open surgical exploration through an anterior midline incision. A trial to bridge the gap between the upper ureter and renal pelvis failed due to the intrarenal nature of the pelvis and the severe adhesion, and indeed, there was a small injury of the IVC during dissection. So, with huge difficulty, a ureterocalyceal anastomosis was finally performed to another calyx (one of the anterior calyces) despite the nondilated nature of the calyces, previous surgery in the area, and the reasonably thick renal parenchyma as he totally refused the idea of nephrectomy. This required resection of a small wedge of renal tissues to prevent future anastomotic stenosis. Postoperatively, he did well and was discharged on the 7th postoperative day.

## 3. Discussion

Postoperative urinary leak following partial nephrectomy is a well-documented complication [6, 8]. The majority of these cases present as persistent discharge from the retroperitoneal drain, nephrocutaneous fistula, urinary collection, abdominal symptoms, or systemic manifestation [6–8]. The management of urinary leak depends on the presentation, extent of leak, and the status of the patient and includes spontaneous resolution, retrograde ureteral stents insertion, insertion of a nephrostomy tube, or percutaneous drainage of the perirenal urinoma or even nephrectomy [6, 7]. This is the first case of urinary leak postlaparoscopic partial nephrectomy which did not heal despite several attempts of drainage and led to the formation of a ureterocalyceal fistula.

In the current case, it is difficult to ascertain the cause of the stenosis of the upper ureter but it appears that a combination of urinary leak at the site of partial nephrectomy and a possible ureteric injury had resulted in the formation of the ureterocalyceal fistula. The most likely cause for the expected ureteric injury was an inadvertent diathermy injury to the vascular supply of the ureter or even a direct but unnoticed ureteric injury as the tumor was relatively close to the ureter. The fact that no direct injury was obvious during laparoscopic dissection and the fact that the retrograde pyelography and the insertion of JJ stent were totally uneventful when performed almost a week after surgery indicate that the injury was a mild one which took some time to cause a break in the ureteric wall and fistula formation. One of the factors that could have caused or at least contributed to the ureteric injury is the infections and collections, which formed postoperatively in the adjacent area. Other remote possibility was the inadvertent injury by the percutaneous needle during drainage of the perirenal collection which was located at the lower pole and upper ureter.

In this case, no glue was used during the procedure. In this regard, there was a report of urine leak associated with fibrin glue which led to almost complete occlusion and stricture formation of the upper ureter which was associated with enfolding of the ureter by fibrin glue causing marked stiffness and adhesions [9].

The early removal of the JJ stent due to severe bladder spasms and intolerance despite the use of anticholinergics must have also contributed to the formation of the fistula. If the stent was kept in for longer time, this probably would have ended in ureteric stricture but prevented this complication.

In conclusion, herein we report on the first case of a ureterocalyceal fistula following laparoscopic partial nephrectomy of an inferiomedial renal tumor. Careful dissection of the tissues in the medical aspect adjacent to the ureter and leaving a JJ stent for an adequate period in case of suspicion of ureteric injury might prevent such complication.

## Abbreviations

JJ stent: Double J stent.
